# White matter abnormalities are also repeatedly present in patients with systemic mast cell activation syndrome

**DOI:** 10.1038/s41398-018-0143-5

**Published:** 2018-05-10

**Authors:** B Haenisch, GJ Molderings

**Affiliations:** 1 German Center for Neurodegenerative Diseases (DZNE), Bonn, Germany; 2 Institute of Human Genetics, University Hospital of Bonn, Bonn, Germany

In their article entitled “Neuroimaging evidence of brain abnomalities in mastocytosis” Boddaert et al.^1^ demonstrated in a pilot study that 49% of 39 systemic mastocytosis (a primary mast cell disease) patients with neuropsychiatric complaints had morphological brain abnormalities, mainly abnormal punctated white matter abnormalities (WMA). In addition, patients with WMA showed increased perfusion in the putamen compared with patients without WMA and with healthy controls (although unfortunately the prevalence of WMA in the healthy controls was not reported). The authors suggest that these morphological and functional brain abnormalities could be caused by the systemic mastocytosis and might be related to neuropsychiatric symptoms of systemic mastocytosis patients.

Primary mast cell disease, i.e., a disease due to pathologically altered mast cells, has long been thought to be just the one rare proliferative disease of systemic mastocytosis with its several subtypes^2^. Suspicion of rather prevalent disease (up to 17%)^3^ of primary aberrant mast cell activation and only limited proliferation, now termed mast cell activation syndrome (MCAS), arose a decade ago. Similar mutational menageries have been detected in systemic mastocytosis and MCAS^4^ engendering the term of mast cell activation disease (MCAD). Thus, MCAD comprises a heterogeneous group of multifactorial, polygenic (genetic and epigenetic) disorders^5^ characterized by aberrant release of variable subsets of up to 200 different mast cell mediators together with accumulation of either morphologically altered and immunohistochemically identifiable mutated mast cells due to mast cell proliferation (systemic mastocytosis and mast cell leukemia), or morphologically ordinary mast cells due to decreased apoptosis (MCAS and well-differentiated systemic mastocytosis).

**Fig. 1:**
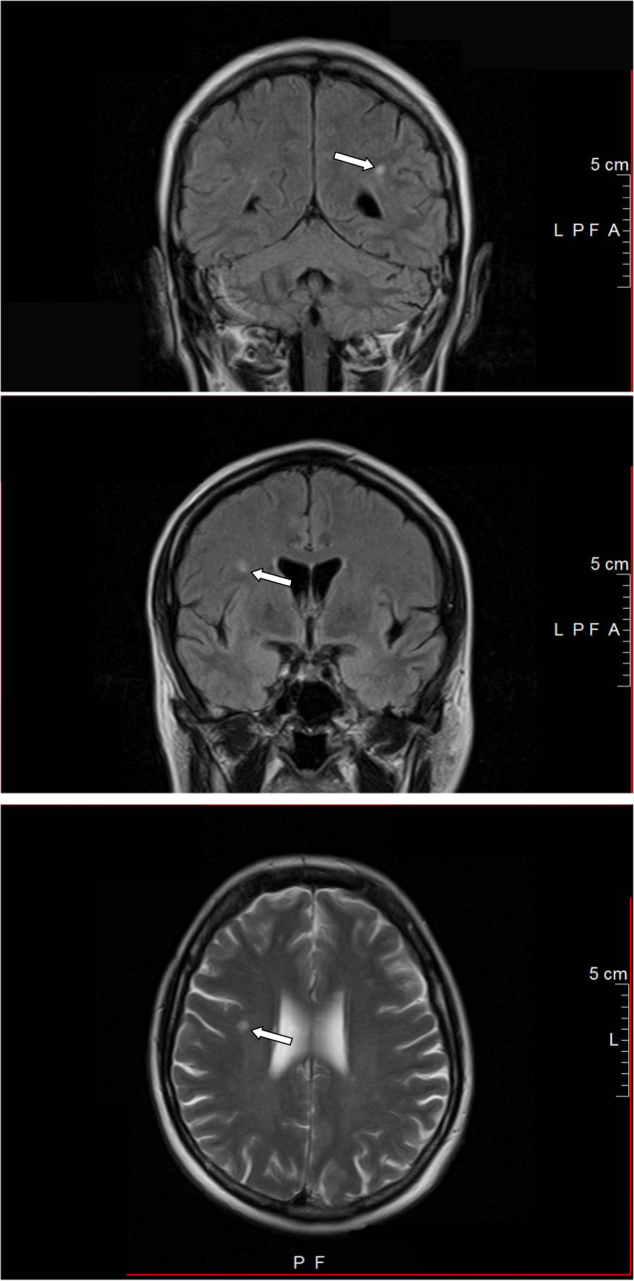
Selected punctate white matter abnormalities (see arrows) in a 55-year-old female patient with systemic mast cell activation syndrome with neurological symptoms (“fibromyalgia phenotype”^2^). The number of WMAs (one WMA first detected in the year 2005) has increased in the course of the disease. The magnetic resonance tomography pictures shown were made in the year 2014

The investigation of the prevalence of WMA in patients with the common variant of MCAD, i.e., MCAS, would be the logical completion of the study by Boddaert et al. which, however, has not yet been performed. Our Interdisciplinary Multicenter Research Group on Mast Cell Diseases Bonn is specialized in diagnosing MCAS according to current criteria^2^. Although we did not yet analyze systematically the occurrence of WMA in our more than 500 MCAS patients (a subset of the cohort has been characterized in ref. ^6^, in whom reasons for secondary mast cell activation were excluded using appropriate assessments, including laboratory testing, imaging, and in all patients endoscopy) we have seen similar alterations in the brain (Fig. 1) as reported by Boddaert et al. for mastocytosis patients repeatedly in our MCAS patients with neuropsychiatric symptoms. Also in our MCAS patients a causative relation between the WMA and the neurological symptoms was not conclusive. Nevertheless, we strongly support the authors´ suggestion that large systematic studies on morphological and functional brain abnormalities in mastocytosis patients are urgently needed because of their potential great clinical relevance.
